# Evaluating the Correct Documentation of Physician Details and Time in General Surgery Ward Round Notes: An Audit and Re-Audit

**DOI:** 10.7759/cureus.98900

**Published:** 2025-12-10

**Authors:** Ahmed Al-Wizni

**Affiliations:** 1 General Surgery, Broomfield Hospital, Mid and South Essex NHS Foundation Trust, Broomfield, GBR

**Keywords:** audit and re-audit cycle, audit cycle, clinical documentation audit, clinical documentation improvement, current guidelines, general surgery, medical record keeping, quality improvement research, re-audit, ward round

## Abstract

Background

Accurate medical documentation is essential for patient safety, continuity of care, and medico-legal accountability. Incomplete or inconsistent entries can compromise communication within the multidisciplinary team and expose healthcare providers to legal risk. This audit assessed compliance of general surgery ward round documentation with General Medical Council (GMC), Royal College of Surgeons, and local Trust standards, focusing on the inclusion of the date, time, clinician’s full name, signature, and GMC number.

Methodology

The audit was conducted across three general surgery wards at Broomfield Hospital. The initial audit data were collected on March 27-28, 2025, and the re-audit data were collected on June 26-27, 2025, three months later. Ward round notes were reviewed for compliance with the following five key parameters: date, time, full name, signature, and GMC number. Following presentation of the initial findings, targeted interventions were implemented, including educational posters, email reminders, and encouragement for doctors to use personal stamps. Data were analysed using Microsoft Excel, and chi-squared testing was used to determine statistical significance at p-values <0.05.

Results

In total, 83 ward round notes were analysed in the initial audit cycle and 92 in the re-audit. Compliance improved across all parameters, i.e., date (100%), time (27.7%→90.2%), full name (67.5%→87.0%), signature (72.3%→94.6%), and GMC number (12.0%→75.0%), with statistically significant improvements in all except the date parameter (p < 0.05).

Conclusions

Targeted awareness and educational interventions significantly improved adherence to documentation standards within the general surgery department. These measures enhanced record-keeping accuracy, improved professional accountability, and may help reduce medico-legal risk and associated financial costs to the Trust.

## Introduction

Comprehensive and accurate medical record-keeping is essential for maintaining effective surgical care. Accurate documentation of a patient’s records provides the progress of a patient and serves as an important form of communication among members of the multidisciplinary team to maintain high levels of care and continuity of care [1,2]. Accurate documentation is also important for providing legal protection for those involved in the care of the patient and contributes to quality assurance and audit processes [3].

The General Medical Council (GMC) and Royal College of Surgeons (RCS) recommend that every clinical entry should include the following features to maintain accuracy: date, time, full name, signature of clinician, and GMC number of the authoring clinician [4-6]. In practice, adherence to these standards varies significantly, particularly in high-pressure environments such as a fast-paced surgical ward round where multiple members of the multidisciplinary team are involved in contributing to the notes [7,8]. Although prior audits have focused on documentation in emergency departments, less attention has been placed on assessing the correct documentation quality during inpatient surgical ward rounds, a setting often impacted by suboptimal record-keeping. Poor documentation practices have also been shown to negatively affect data quality, communication, and overall clinical workflow across hospital settings [9,10]. Inadequate or missing documentation has been associated with increased clinical risk and substantial financial implications for healthcare providers, contributing to millions in medico-legal costs annually within the NHS [11,12].

Therefore, this audit was conducted within the Department of General Surgery at Broomfield Hospital to evaluate compliance with GMC, RCS and local Trust documentation standards for general surgery ward round notes, and to assess the impact of a structured educational intervention on improving documentation accuracy.

## Materials and methods

Audit design

The audit was conducted across three general surgery wards (Heybridge, Rayne, and SEW) over two consecutive days. Each ward round entry was reviewed to determine whether it included the required documentation parameters, i.e., date, time, full name, GMC number, and signature of the documenting clinician. Exclusion criteria included entries for patients in high-dependency or intensive care units and patients not yet admitted at the time of data collection.

Data collection

Data for the initial audit were collected on March 27-28, 2025, and the re-audit was conducted on June 26-27, 2025, approximately three months later. Only documentation parameters were recorded; no patient-identifiable or clinical information was collected. Ward round notes were assessed for inclusion of the date, time, full name, GMC number, and clinician signature.

Standards

According to GMC and RCS guidance, clinical documentation should include all five parameters (date, time, full name, GMC number, and signature). In line with national audit expectations, a target compliance threshold of 90% was used to benchmark acceptable performance [4-6].

Intervention

**Figure 1:**
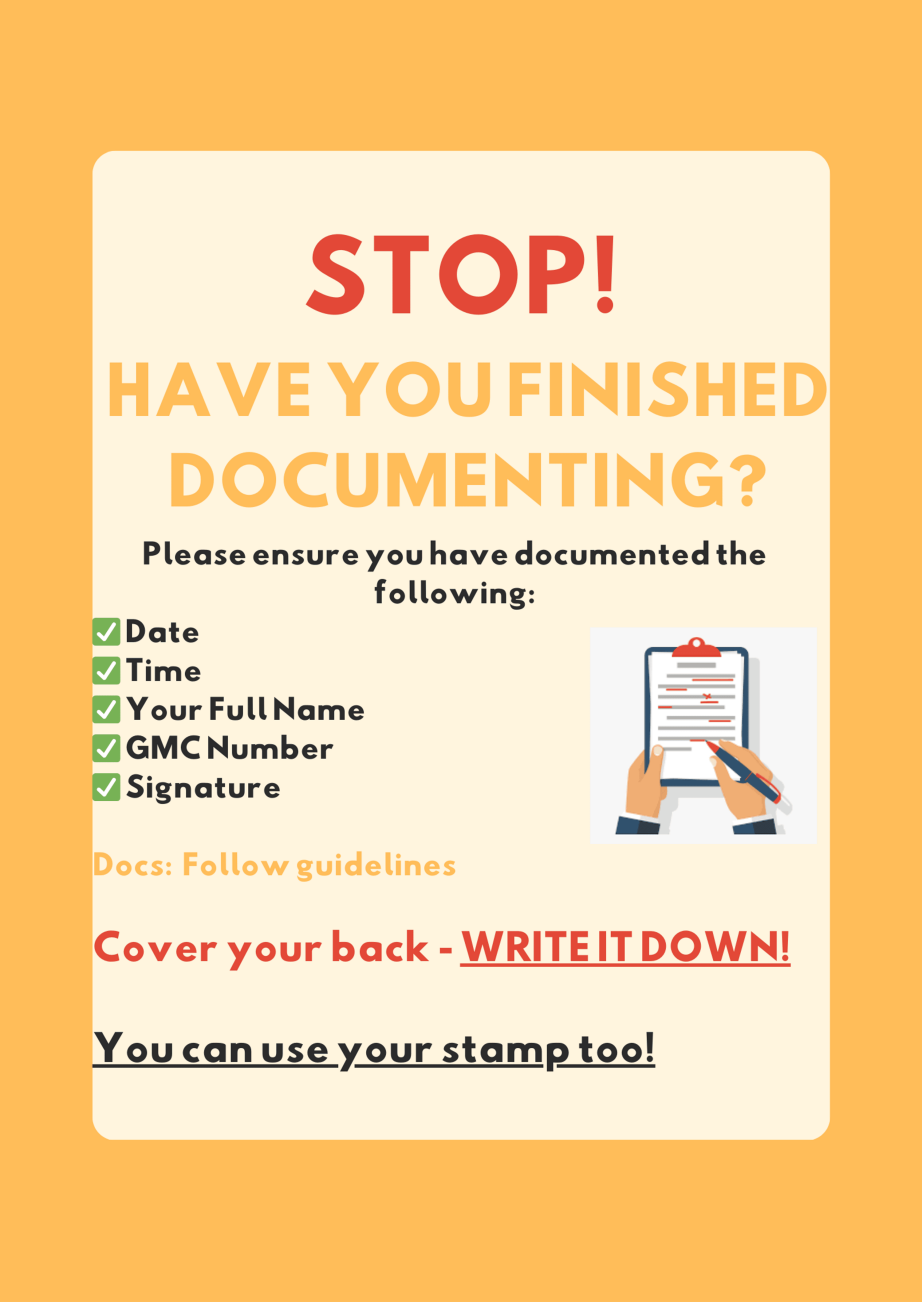
Awareness poster posted in wards, ward offices, and doctors’ offices. Created using Canva. Author’s own image, Broomfield Hospital General Surgery Department.

Following the initial analysis, findings were presented at the General Surgery Department Audit Meeting to present awareness of documentation deficiencies relative to national standards. Educational awareness posters were developed and displayed to summarise the correct documentation requirements (Figure 1). These were displayed in ward offices, doctors’ rooms, and the wards where ward rounds were conducted. Reminder emails were also circulated, and clinicians were encouraged to use personalised stamps containing their full name and GMC number to facilitate efficient and accurate documentation during fast-paced ward rounds.

Re-audit

Approximately three months after the interventions were implemented, the same wards were re-assessed using the same methodology. All data were entered and analysed using Microsoft Excel, and statistical significance between audit cycles was determined using chi-squared testing with a significance level of p < 0.05. Results were then presented at the General Surgery Departmental Audit Meeting.

## Results

Audit

**Figure 2:**
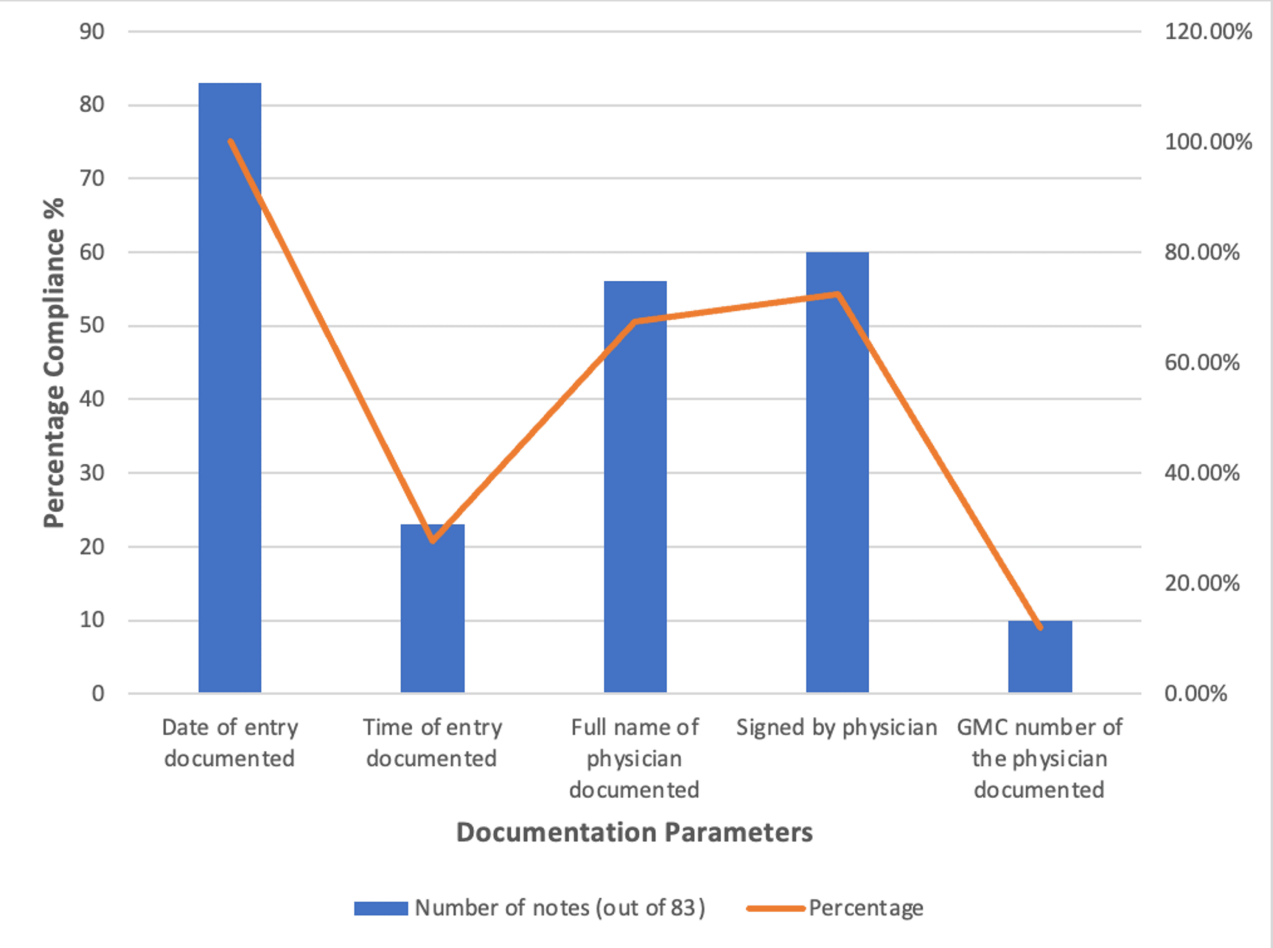
Baseline audit compliance for documentation parameters in general surgery ward round notes.

Of the total 113 patient ward round notes, 30 (26.5%) were excluded due to patients being in intensive therapy unit (ITU)/high-dependency unit (HDU)/home/not yet admitted. Following exclusion, the initial audit included 83 eligible ward round notes. Statistical analysis of the 83 General Surgery ward round medical records was conducted for the audit. Results demonstrated that all the documented ward round notes recorded the date of entry (100%) due to the prefilled dated ward round sheet that is printed every morning before ward rounds (Figure 2). The second highest compliant parameter was the signature of the authoring clinician, with 72.3% (60/83) compliance. Full name had a compliance of 67.5% (56/83). However, the time of entry and the GMC number of the clinician were very low, documented in 27.7% (23/83) and 12.0% (10/83) of entries, respectively.

Re-audit

**Figure 3:**
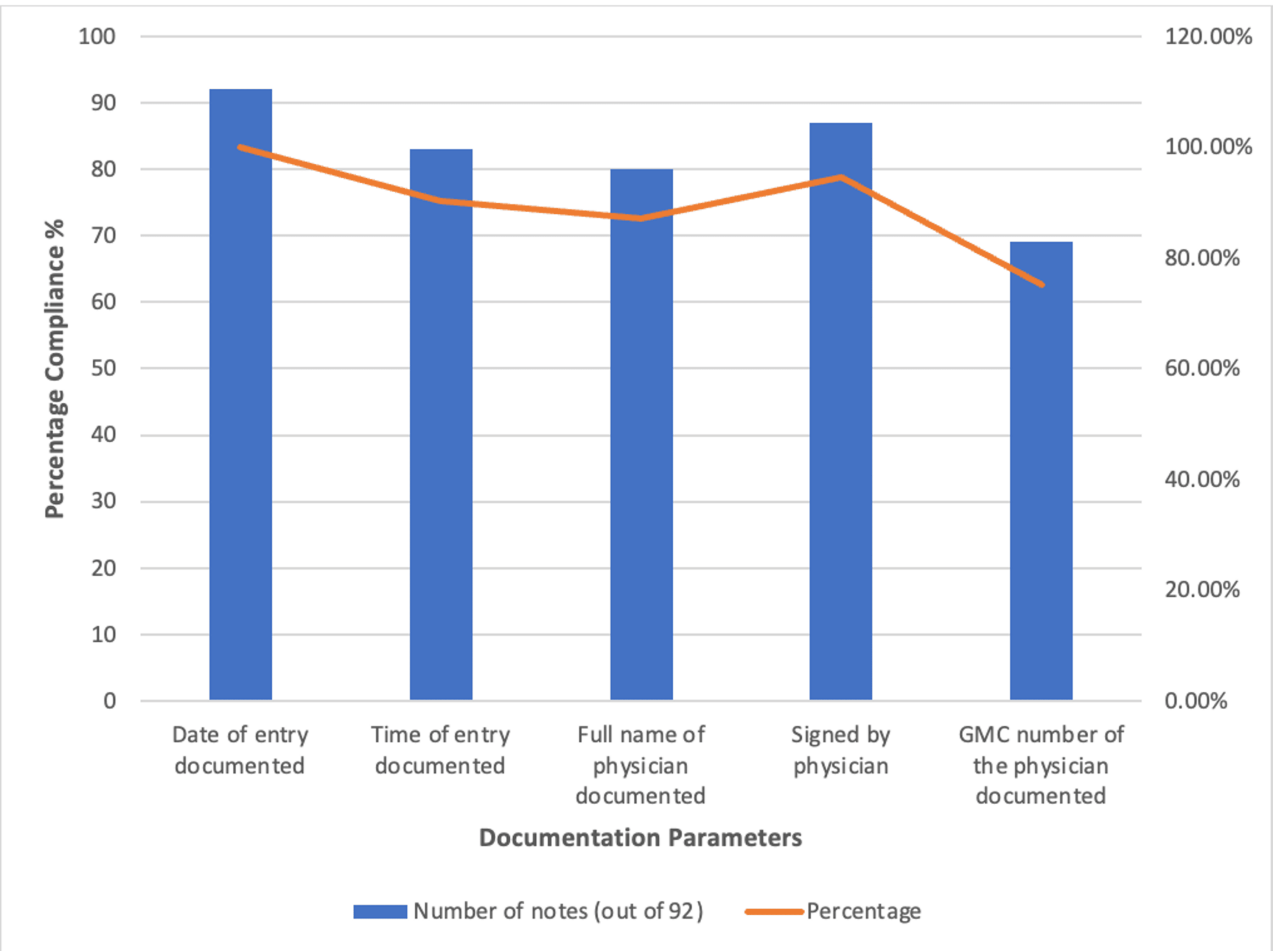
Re-audit compliance for documentation parameters after three months of the intervention.

A total of 109 patient ward round notes were collected, of which 17 (15.6%) were excluded due to patients being in ITU/HDU, at home, or not yet admitted. Following exclusions, 92 ward round notes remained for statistical analysis in the re-audit. Results demonstrated improvement across all parameters following the intervention, while the date remained at 100% (92/92) due to the prefilled proforma (Figure 3). Excluding this, the highest compliant parameter was the clinician’s signature, documented in 94.6% (87/92) of entries. Time of entry was documented in 90.2% (83/92), followed by full name in 87.0% (80/92). The least compliant parameter was the GMC number, which improved to 75.0% (69/92), representing a substantial increase compared with the initial audit.

The statistical level of significance between the number of ward round medical records documenting each parameter was set to 0.05 and evaluated using the chi-squared test. There was a significant improvement seen in the time of entry documented, the full name of the clinician documented, the signature, and the GMC number documented (p < 0.01) (Table 1). However, there was no significant difference in the date of entry parameter due to being at 100% in both the audit and re-audit (p = 1).

**Table 1:** Comparison of documentation compliance between audit cycles with the chi-squared analysis.

Parameter	Audit (n = 83)	Re-audit (n = 92)	Chi-square value	P-value	Significant (p < 0.05)
Date of entry documented	83 (100%)	92 (100%)	0.000	1.000	No
Time of entry documented	23 (27.7%)	83 (90.2%)	68.79	<0.001	Yes
Full name of clinician documented	56 (67.5%)	80 (87.0%)	8.48	0.0036	Yes
Signed by clinician	60 (72.3%)	87 (94.6%)	14.50	<0.001	Yes
GMC number documented	10 (12.0%)	69 (75.0%)	67.31	<0.001	Yes

## Discussion

The acceptable target for each parameter being included in ward round notes was 100%. This audit revealed that the majority of general surgery ward round notes, including the five parameters of date of entry, time, full name, signature, and GMC number, results remained suboptimal. However, a significant improvement was seen after implementing simple awareness strategies, with compliance improving across all areas. These findings highlight how relatively low-cost interventions can influence clinical behaviour and documentation quality, ultimately reducing medico-legal costs for the trust through improved accuracy of ward round notes. Incomplete or inaccurate clinical documentation is a well-recognised cause of litigation and financial loss within the NHS, where poor record-keeping contributes significantly to negligence claims and settlements [9,10].

The significant improvements seen in the parameters tested are consistent with evidence from other settings where educational interventions improved adherence to documentation standards [7,8]. As a result of incomplete documentation, reduced accountability, communication breakdown and possible medico-legal challenges may occur [11,12]. In the setting of general surgery, accurate records are particularly crucial as the operative and postoperative management plans are influenced by the ward round records.

In general surgery, the ward round can be extremely high-paced and sometimes compliance to five parameters, although important to include, can be timely to include, especially with handwritten paper medical records. Often it can be a stressful and busy environment where patients require urgent medical management and need to be seen efficiently with the consultant who needs to see numerous patients before going into the theatre to operate. The authoring clinician must also be thorough when noting the important factors when patients are seen during the ward round with the rest of the multidisciplinary team that includes the consultant, registrar, resident doctor, physician associates, nurses, and other members of the team. In such an intense environment, it can often be easy to neglect correct documentation when comparing these factors to the rest of the documentation of ward round records; they can often be seen as less important and therefore overlooked. Regardless, it must be noted that it is still an essential part of the ward round records to maintain best medical practice and maintain good levels of communication between responsible healthcare professionals. Attention must be paid to ensure these features are included in all surgical and medical ward round records to act as valuable legal evidence if needed.

Two of the lowest parameters seen being included in ward round notes were time of entry and GMC number in the initial audit, whereas in the re-audit, the two lowest were full name and GMC number. Both the audit and re-audit had the lowest compliance for the GMC number. Possibly reinstating that the lowest compliant parameter may be due to the negligence of this, being the most time-consuming to include, and some may not have it memorised, which increases the time needed to include. To improve this parameter, in the initial presentation at the General Surgery Audit Meeting, doctors were encouraged to obtain personalised stamps that included their full name and GMC number. Many doctors had implemented this change in their practice, as seen when collecting the data for the re-audit, although many still had not, and while some of these had committed the GMC number to memory and handwritten it when documenting, some still did not. Therefore, it was suggested to the department lead whether approval for funding could be obtained to get individualised stamps for all resident doctors starting in general surgery every rotation. This was received well by the general surgery lead consultant, and as a direct consequence of the results of the audit and re-audit, stamps for doctors are now being implemented to improve the accuracy and consistency of documentation. The department lead consultant also valued the project highly and has committed to making this a recurring project for every rotation, embedding it as a standing departmental audit going forward.

To maintain sustained improvement of documentation moving forward, record-keeping standards should be upheld and reinforced throughout each newly rotating resident doctor through education during departmental induction and ongoing teaching. Providing personalised stamps would improve ease and convenience, and adopting electronic record systems could also further enhance compliance as well as clarity.

## Conclusions

The audit assessed documentation standards compliance with GMC and RCS guidelines within the General Surgery Department with including date, time, full name, signature of clinician, and GMC number. Implementation of targeted awareness and education measures significantly improved documentation standards and led to changes within the department, such as implementing stamps and changes to induction for newly rotating doctors that encourage and lead to improvements in documentation standards and adherence with guidelines. These interventions not only enhanced adherence to documentation standards but also have the potential to reduce medico-legal risk and associated financial costs to the Trust. Embedding documentation education into routine practice and introducing these changes may sustain and expand these improvements.
